# Free-standing membrane incorporating single-atom catalysts for ultrafast electroreduction of low-concentration nitrate

**DOI:** 10.1073/pnas.2217703120

**Published:** 2023-03-06

**Authors:** Xiaoxiong Wang, Xuanhao Wu, Wen Ma, Xuechen Zhou, Shuo Zhang, Dahong Huang, Lea R. Winter, Jae-Hong Kim, Menachem Elimelech

**Affiliations:** ^a^Institute for Ocean Engineering, Shenzhen International Graduate School, Tsinghua University, Shenzhen 518055, China; ^b^Department of Chemical and Environmental Engineering, Yale University, New Haven, CT 06520; ^c^Department of Environmental Engineering, Zhejiang University, Hangzhou 310058, China; ^d^Department of Chemical and Biotechnology Engineering, Université de Sherbrooke, Sherbrooke, QC J1K 2R1, Canada; ^e^Department of Civil and Environmental Engineering, The Pennsylvania State University, University Park, PA 16802; ^f^College of Environmental Science and Engineering, Ministry of Education Key Laboratory of Pollution Processes and Environmental Criteria, Tianjin Key Laboratory of Environmental Remediation and Pollution Control, Nankai University, Tianjin 300350, China; ^g^School of Environment and Civil Engineering, Dongguan University of Technology, Dongguan, Guangdong 523808, China

**Keywords:** low-concentration nitrate reduction, free-standing electrified membrane, single-atom catalyst, carbonaceous interwoven structure, activity and selectivity improvement

## Abstract

The release of wastewaters containing relatively low levels of nitrate (NO_3_^−^) results in sufficient contamination to induce harmful algal blooms and to elevate drinking water NO_3_^−^ concentrations to potentially hazardous levels. In particular, the facile triggering of algal blooms by ultra-low concentrations of NO_3_^−^ necessitates the development of efficient methods for NO_3_^−^ destruction. However, promising electrochemical methods suffer from weak mass transport under low reactant concentrations, resulting in long treatment times (on the order of hours) for complete NO_3_^−^ destruction. In this study, we present flow-through electrofiltration via an electrified membrane incorporating nonprecious metal single-atom catalysts for NO_3_^−^ reduction activity enhancement and selectivity modification, achieving near-complete removal of ultra-low concentration NO_3_^−^ (10 mg-N L^−1^) with a residence time of only a few seconds (10 s). By anchoring Cu single atoms supported on N-doped carbon in a carbon nanotube interwoven framework, we fabricate a free-standing carbonaceous membrane featuring high conductivity, permeability, and flexibility. The membrane achieves over 97% NO_3_^−^ removal with high N_2_ selectivity of 86% in a single-pass electrofiltration, which is a significant improvement over flow-by operation (30% NO_3_^−^ removal with 7% N_2_ selectivity). This high NO_3_^−^ reduction performance is attributed to the greater adsorption and transport of nitric oxide under high molecular collision frequency coupled with a balanced supply of atomic hydrogen through H_2_ dissociation during electrofiltration. Overall, our findings provide a paradigm of applying a flow-through electrified membrane incorporating single-atom catalysts to improve the rate and selectivity of NO_3_^−^ reduction for efficient water purification.

## Significance

The release of nitrate-containing wastewaters leads to eutrophication of aquatic ecosystems and contamination of drinking water sources. Electrochemical methods for NO_3_^− ^destruction are limited by expensive materials, by-product ammonia generation, and weak mass transport. Here, we employ an electrified membrane incorporating single-atom catalysts to modify the kinetics and pathways of NO_3_^− ^reduction. We provide a method for trapping electrocatalysts with specific structure into a carbon nanotube interwoven framework for free-standing membrane fabrication. In addition to intensifying mass transport, electrofiltration enhances adsorption of the intermediate reduction species to the catalysts and improves their interactions with hydrogen species to promote N_2_ formation. These mechanisms enable orders-of-magnitude enhancement of NO_3_^−^ reduction rate with superior N_2_ selectivity without requiring precious metals.

Eutrophication, caused by the enrichment of nutrients such as nitrate (NO_3_^−^), threatens water resources and aquatic ecosystems (1, 2). Globally, the emergence of harmful algal blooms has increased since the 1980s (3) due to the excessive application of fertilizers and the improper discharge of nutrient-containing wastewaters (4, 5). Algal blooms can be triggered at ultra-low NO_3_^−^ levels of 1 to 2 mg-N L^−1^ in oligotrophic waters, (6, 7) whereas NO_3_^−^ concentration limits for water disposal in countries such as the United States and China are usually higher than 10 mg-N L^−1^ (8, 9). When these concentrated streams are released to water bodies, the NO_3_^−^ concentration becomes sufficient for algae growth, especially near wastewater discharge points. Elevated NO_3_^−^ levels in aquatic environments also lead to contamination of drinking water sources, resulting in human health concerns related to endocrine disruption and carcinogenicity. (10) However, efficient methods for near-complete conversion of NO_3_^−^ are limited. Mass transport limitations under low concentrations necessitate a long residence time of hours to completely remove NO_3_^−^ from water, whether applying biological or catalytic methods (11–13).

Electrochemical reduction of NO_3_^−^ to nitrogen gas (N_2_) or ammonia (NH_3_), which involves mild operating conditions and does not require external chemical input, offers an environmentally friendly approach for NO_3_^−^ removal. (14–17) NH_3_ recovery from wastewaters via NO_3_^−^ reduction could provide a sustainable alternative to offset fossil fuel-intensive conventional NH_3_ synthesis. (18) However, the economic feasibility of NH_3_ recovery in diverse waste streams remains uncertain, especially given the need for subsequent separation and conversion of dilute ammonium in mixed waste streams to useful forms. Given the current widespread release of nitrate-containing wastewaters to the environment, efficient methods for reducing NO_3_^−^ to environmentally benign N_2_ should be more viable in cases where NH_3_ recovery may not be economical or practical. Nevertheless, high reduction selectivity to N_2_ has been difficult to achieve at competitive treatment cost at present due to the need for precious platinum group metals. (19, 20)

Single-atom catalysts have exhibited superior activity toward the electrochemical NO_3_^−^ reduction reaction (NO_3_RR). (21–23) The maximized utilization of the metal atoms results in a high surface area of active sites as well as strong interactions between the atoms and the support material to enable efficient charge transfer. (24–26) Copper single-atom (Cu_1_) catalysts with copper−N_4_ moiety-doped carbon (Cu−N_4_/C) have been identified to be highly active for the electrochemical NO_3_RR. (27, 28) However, copper-based electrocatalysts generally provide low N_2_ selectivity because of the mismatch between the concentration of the intermediate reduction species (mainly nitric oxide, NO) and the supply of hydrogen (mainly atomic hydrogen, H*) for N_2_ formation. (29–31) Additionally, the reaction rate of NO_3_RR under conventional flow-by operation mode is limited by weak mass transport, resulting in a small concentration gradient near the electrode surface. (32, 33)

Incorporating catalysts with high NO_3_RR activity into electrified membranes (EMs) for flow-through operation could potentially address the challenges associated with limited reaction rate and N_2_ selectivity when treating low concentrations of NO_3_^−^. Advection through EMs with high electrode surface areas can intensify mass transport and maximize catalyst utilization efficiency. (34) The efficient utilization of in situ generated H* (35) and the enrichment of the reactants in confined membrane pores (36) could also potentially modify the selectivity to N_2_. Additionally, considering their dual functions of electroactivity and solute separation, EMs could be easily integrated into existing systems or serve as point-of-use devices for water treatment. (32, 37) To date, however, few fabrication methods are available for directly incorporating catalysts with specific structure such as single-atom catalysts into EMs for NO_3_^−^ removal.

Herein, we develop a free-standing carbon nanotube (CNT) EM incorporating Cu_1_ anchored on N-doped carbon (Cu_1_/NC@CNT-FEM) for highly efficient and selective reduction of ultra-low concentration NO_3_^−^. We trap the Cu_1_ catalysts with a Cu−N_4_/C structure in a CNT interwoven framework to form the free-standing carbonaceous membrane with high electrical conductivity, water permeability, and flexibility. The membrane can achieve near-complete reduction of 10 mg-N L^−1^ NO_3_^−^ with high N_2_ selectivity in a single-pass electrofiltration with a residence time of only a few seconds. We further investigate the mechanisms involved in rate acceleration and selectivity modification for the electrochemical NO_3_RR using the Cu_1_/NC@CNT-FEM, including the effects of H* generation and intermediate NO adsorption during flow-through operation. Our findings show that the application of EMs incorporating single-atom catalysts could enable efficient purification of water with ultra-low NO_3_^−^ concentration without the use of precious metals.

## Results

### Fabrication and Characterization of Cu_1_/NC@CNT-FEM.

Cu_1_ anchored on N-doped carbon (Cu_1_/NC) catalysts were synthesized via pyrolysis of Cu-doped ZIF-8 precursors at 900 °C under argon atmosphere. Scanning electron microscopy (SEM) shows a rhombic dodecahedral morphology of the catalysts (*SI Appendix*, Fig. S1). No characteristic peaks can be assigned to Cu-related crystal phases in X-ray diffraction (XRD) patterns (*SI Appendix*, Fig. S2), consistent with the lack of observable nanoparticles using transmission electron microscopy (TEM) characterization (*SI Appendix*, Fig. S1). The X-ray photoelectron spectroscopy (XPS) peak for Cu 2p_3/2_ at 935.1 eV suggests a valence state of Cu in the Cu_1_/NC close to +2 (*SI Appendix*, Fig. S3). Additionally, peaks indexed to pyridinic, pyrrolic, and graphitic N species are found in the N 1s spectrum of XPS.

**Fig. 1.**
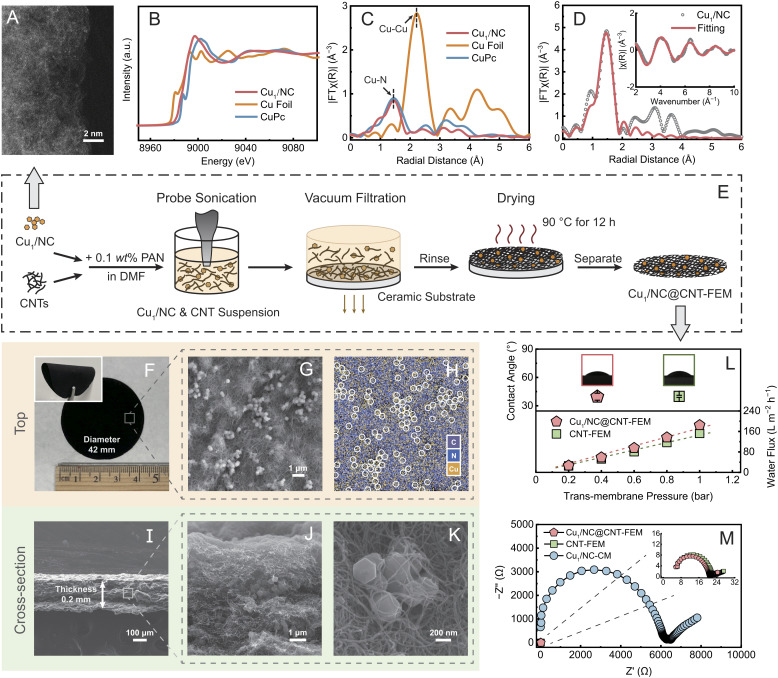
Characterization of copper single-atom anchored on N-doped carbon (Cu_1_/NC) catalysts and free-standing electrified CNT membrane incorporating Cu_1_/NC (Cu_1_/NC@CNT-FEM). (*A*) HAADF-STEM image of Cu_1_/NC. (*B*) Normalized Cu *K*-edge XANES and (*C*) FT-EXAFS spectra of the Cu_1_/NC, copper foil (Cu-Cu reference), and copper phthalocyanine (CuPc, Cu-N reference). (*D*) Fitting of the Cu_1_/NC FT-EXAFS spectrum. *Inset* is the corresponding *K*-space spectrum. (*E*) Schematic illustrating the fabrication procedure of the Cu_1_/NC@CNT-FEM. (*F*) Photographs of the Cu_1_/NC@CNT-FEM. The *Inset* shows the folded membrane. (*G*) SEM image of the *Top* view of the Cu_1_/NC@CNT-FEM. (*H*) EDS mapping images (overlapped with C, N, and Cu elements) of the Cu_1_/NC@CNT-FEM. White circles indicate the areas where the Cu_1_/NC are visible in *G*. SEM images of the cross-sectional view of the Cu_1_/NC@CNT-FEM at (*I*) low and (*J*) high magnifications. (*K*) SEM image depicting the Cu_1_/NC bound in the CNT interwoven structure. (*L*) Water contact angles (*Top*) and water flux (*Bottom*) of the Cu_1_/NC@CNT-FEM and a free-standing electrified CNT membrane (CNT-FEM) without incorporating Cu_1_/NC. (*M*) EIS spectra of the Cu_1_/NC@CNT-FEM, the CNT-FEM, and a Cu_1_/NC functionalized ceramic membrane (Cu_1_/NC-CM) over a frequency range of 1 to 10^6^ Hz in 10 mM Na_2_SO_4_ solution.

High-angle annular dark-field scanning transmission electron microscopy (HAADF-STEM) characterization suggests the presence of atomically dispersed Cu on Cu_1_/NC (Fig. 1*A*, see enlarged image in *SI Appendix*, Fig. S4*A*). Bright spots in the HAADF-STEM images are only observed on an angstrom scale, suggesting the presence of atomic-scale Cu particles. X-ray energy dispersive spectroscopy (EDS) mapping analysis shows a homogeneous distribution of the Cu atoms on the catalyst (*SI Appendix*, Fig. S4). The lack of metal clusters is further confirmed by Cu *K*-edge X-ray absorption near-edge structure (XANES). The white-line intensity of Cu_1_/NC is similar to that of the copper phthalocyanine (CuPc) reference but very different from the copper foil reference, suggesting that the valence state of the Cu atoms is around +2 (Fig. 1*B*). The Fourier-transformed extended X-ray absorption fine structure (FT-EXAFS) spectrum of Cu_1_/NC exhibits a main peak at a radial distance of 1.44 Å (Fig. 1*C*), overlapping with the Cu−N interaction in the CuPc reference. The lack of a peak related to Cu−Cu coordination at ~2.2 Å suggests the absence of metal clusters, further supporting a Cu structure consisting of dispersed single atoms. The fitted FT-EXAFS spectrum shows a Cu−N coordination number of 4.4 ± 0.4 (Fig. 1*D* and *SI Appendix*, Table S1), indicating a Cu−N_4_/C structure in the Cu_1_/NC.

We demonstrate a method for fabricating a free-standing EM via direct trapping of the catalysts into a CNT interwoven framework. In brief, a Cu_1_/NC and CNT suspension containing polyacrylonitrile (PAN) is blended by probe sonication, followed by vacuum-filtering onto a ceramic substrate with a total material loading of 100 mg (Fig. 1*E*). The free-standing electrified CNT membrane incorporating Cu_1_/NC (Cu_1_/NC@CNT-FEM) is obtained by peeling off the carbonaceous layer from the substrate after rinsing and drying. The membrane (diameter of 42 mm) is uniform and flexible (Fig. 1*F*), which is due to the compact and interwoven CNT configuration (*SI Appendix*, Fig. S5) as well as the added mechanical strength from the PAN binder. As shown in the top-view SEM and EDS mapping images (Fig. 1 *G* and *H*), CNTs constitute the framework of the membrane with an average pore size of 42.4 nm (*SI Appendix*, Fig. S6), and the Cu_1_/NC catalysts are dispersed uniformly in the framework. The cross-section view shows a 0.2-mm thickness of the free-standing membrane (Fig. 1*I*). The catalysts are bound by the CNTs and trapped in the membrane, resulting in stable catalyst immobilization in the membrane (Fig. 1 *J* and *K*).

The introduction of the Cu_1_/NC does not significantly affect the physical and electrical properties of the CNT-based membrane framework. The Cu_1_/NC@CNT-FEM exhibits the same water contact angle (39.6°) as an unmodified free-standing CNT EM (CNT-FEM) (Fig. 1*L*, *Top*), where the small water contact angle results from the addition of hydrophilic PAN in the porous structure. The water permeability of the Cu_1_/NC@CNT-FEM (179 L m^−2^ h^−1^ bar^−1^) and the CNT-FEM (150 L m^−2^ h^−1^ bar^−1^) is also similar (Fig. 1*L*, *Bottom*). Notably, we observe a 300-fold difference in the amplitudes of the semicircles for both Cu_1_/NC@CNT-FEM and CNT-FEM compared with a Cu_1_/NC functionalized ceramic membrane (Cu_1_/NC-CM) in the electrochemical impedance spectroscopy (EIS) spectra (Fig. 1*M*). (The carbonaceous layer without the CNT interwoven structure cannot be separated from the ceramic substate.) These results indicate an insufficient electrical conductivity of the membrane active layer consisting only of the catalysts, due to the interference of doped elements and defects in electron transport through the sp^2^ carbonaceous structure and the high resistance at the grain boundaries.

Overall, the CNT interwoven configuration can encapsulate the Cu_1_/NC catalysts to form a free-standing membrane with high electrical conductivity, water permeability, and flexibility. Although CNTs are the most common conductive materials employed for EM fabrication, (38–40) their structural features have not been fully exploited for versatile catalyst integration strategies. Considering the facile membrane fabrication procedures which do not require high temperature or pressure, our proposed method provides a versatile platform for incorporating other electrocatalysts into CNT-based membranes for electrified water treatment applications.

### Electrochemical Nitrate Reduction Performance.

**Fig. 2.**
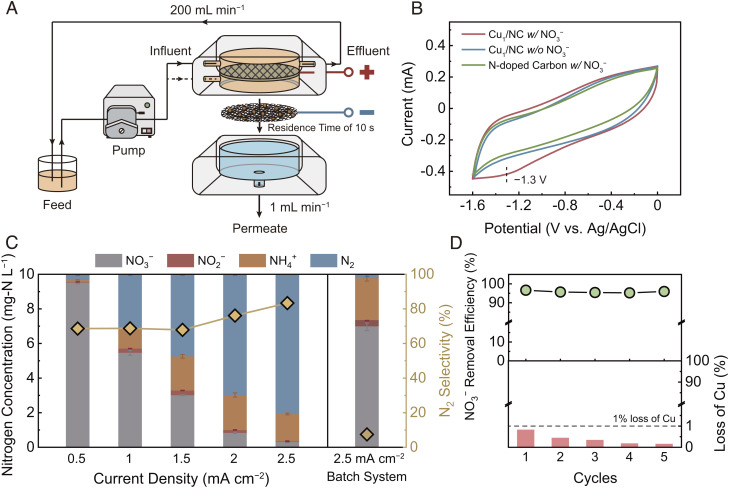
Electrochemical nitrate reduction performance of the Cu_1_/NC@CNT-FEM. (*A*) Schematic illustration of the cross-flow membrane electrofiltration system. The membrane filtration cell contains a feed chamber (orange) with a RuO_2_-IrO_2_/Ti mesh electrode and a permeate chamber (blue). The Cu_1_/NC@CNT-FEM (effective area of 12.6 cm^2^) and the RuO_2_-IrO_2_/Ti mesh serve as the cathode and anode, respectively. Experiments were conducted at a cross-flow rate of 200 mL min^−1^ and a permeate flow rate of 1 mL min^−1^, resulting in a residence time of 10 s in the membrane. (*B*) CV curves of the Cu_1_/NC and N-doped carbon in different electrolytes (100 mM Na_2_SO_4_ or 100 mM Na_2_SO_4_ + 100 mg-N L^−1^ NaNO_3_) using glassy carbon as the support at a scan rate of 20 mV s^−1^. The dashed line indicates the potential where NO_3_^−^ reduction occurs. (*C*) Effect of current density (0.5 to 2.5 mA cm^−2^) on the distribution of nitrogen species in the permeate (left axis) and N_2_ selectivity (right axis) using the Cu_1_/NC@CNT-FEM in the electrofiltration system. The *Right* panel shows the NO_3_^−^ reduction results of the membrane in a batch system at a current density of 2.5 mA cm^−2^. Both filtration and batch experiments were performed by treating 60 mL feed solution with 10 mg-N L^−1^ NaNO_3_ and 10 mM Na_2_SO_4_ for 1 h. Error bars represent SDs from triplicate measurements. (*D*) NO_3_^−^ removal efficiency (*Top*) and loss of Cu (*Bottom*) of the Cu_1_/NC@CNT-FEM as a function of filtration cycles. After operating for 2 h, the membrane was rinsed and dried and then used for the next cycle.

We evaluated the NO_3_^−^ reduction performance of the Cu_1_/NC@CNT-FEM using a cross-flow electrofiltration system with a permeate flow rate of 1 mL min^−1^, as depicted in Fig. 2*A*. We note that the residence time for a single-pass filtration is only 10 s, estimated according to the membrane pore volume and the permeate flow rate. The cyclic voltammetry (CV) curve of the Cu_1_/NC (red) shows a distinct peak at −1.3 V vs. Ag/AgCl, which is absent for the N-doped carbon (green) in an electrolyte containing NO_3_^−^ (Fig. 2*B*). This result suggests that the electrochemical NO_3_RR activity in the membrane is primarily due to the Cu_1_ on the catalysts.

We investigated the effect of current density on NO_3_^−^ reduction in the electrofiltration system using the Cu_1_/NC@CNT-FEM with a feed solution containing 10 mg-N L^−1^ NO_3_^−^ to simulate the concentration of treated wastewaters. As shown in Fig. 2*C*, a clear correlation is observed between the current density and the residual NO_3_^−^ concentration in the permeate after 1-h operation. Notably, the selectivity to N_2_ increases when the NO_3_^−^ concentration in the permeate reaches a relatively low level (0.8 mg-N L^−1^). When increasing the current density to 2.5 mA cm^−2^, the membrane achieves an NO_3_^−^ removal efficiency of 97% (i.e., residual NO_3_^−^ concentration of 0.3 mg-N L^−1^) and a high N_2_ selectivity of 86% within 10 s of residence time in the membrane.

We performed a batch experiment to compare the NO_3_^−^ reduction performance of the Cu_1_/NC@CNT-FEM at the same current density (i.e., 2.5 mA cm^−2^) under flow-by operation mode. The membrane shows only 30% NO_3_^−^ removal in the batch system after 1-h operation (Fig. 2*C*, *Right*), primarily due to the limited mass transport under low NO_3_^−^ concentration. This suggests that electrified flow-through operation can effectively overcome the limitations induced by diffusive transport, resulting in an order-of-magnitude difference of the NO_3_^−^ reduction rate (466 mg-N m^−2^ h^−1^) compared with that obtained in the flow-by mode (45 mg-N m^−2^ h^−1^) when achieving near-complete NO_3_^−^ removal. Additionally, ammonium (NH_4_^+^) is the primary product detected in the batch system (N_2_ selectivity of only 7%). Therefore, the application of the Cu_1_/NC@CNT-FEM for flow-through operation can not only accelerate the NO_3_RR, but it also can modify the selectivity to enhance N_2_ generation over NH_3_.

The incorporation of uniformly dispersed single-atom catalysts in the membrane plays a vital role in maximizing the utilization efficiency of the metal atoms to enhance the NO_3_^−^ reduction performance during electrofiltration. The Cu_1_/NC@CNT-FEM exhibits near-complete NO_3_^−^ removal with a turnover frequency of 1.5 mg-N mg-Cu^−1^ h^−1^, 21% higher than that obtained using Cu nanoparticles incorporated into the CNT-FEM (Cu_NP_/NC@CNT-FEM) (*SI Appendix*, Fig. S7). Further, the intensive blending of the precursors by probe sonication prevents catalyst aggregation and inadequate catalyst binding with the CNTs (*SI Appendix*, Fig. S8), ensuring sufficient dispersion of the catalysts for efficient low-concentration NO_3_^−^ removal (*SI Appendix*, Fig. S9).

The Cu_1_/NC@CNT-FEM maintains a ~100% NO_3_^−^ removal efficiency throughout a long-term testing time of 6 h (*SI Appendix*, Fig. S10). The membrane also exhibits stable NO_3_^−^ reduction performance over multiple electrofiltration cycles with less than 1% loss of the Cu from the catalysts (<3 μg Cu L^−1^ in the treated water, Fig. 2*D*). Additionally, NO_3_^−^ can be removed effectively from simulated surface water (constituents listed in *SI Appendix*, Table S2) and water with low ionic strength (1 mM Na_2_SO_4_) (*SI Appendix*, Fig. S11). These results demonstrate the durability of the membrane for purifying water with ultra-low NO_3_^−^ concentration at near-realistic conditions.

### Mechanism of Nitrate Reduction During Electrofiltration.

**Fig. 3.**
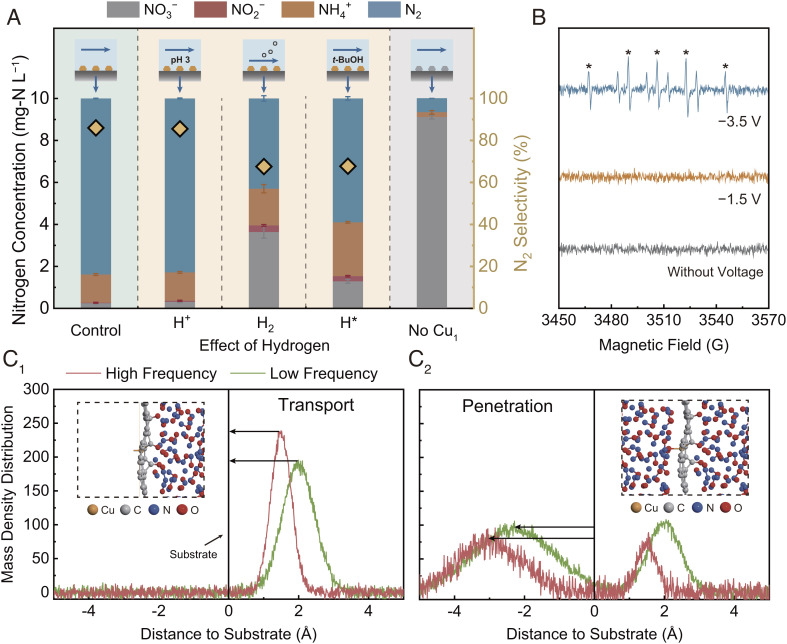
Mechanism investigation for electrochemical nitrate reduction using the Cu_1_/NC@CNT-FEM. (*A*) Effect of modifying the relative contributions of various hydrogen species, including hydrogen ion (H^+^), hydrogen gas (H_2_), and atomic hydrogen (H*), on nitrogen speciation (left axis) and N_2_ selectivity (right axis) using the Cu_1_/NC@CNT-FEM. Investigation of the H^+^, H_2_, and H* species was performed by lowering the feed solution pH to 3.0, applying surface flushing to remove the electrogenerated hydrogen bubbles, and injecting *t*-BuOH (200 mM) into the feed for radical quenching, respectively. The *Left* (green shading) and *Right* (gray shading) panels show the nitrogen reduction results obtained using the Cu_1_/NC@CNT-FEM or a CNT-FEM incorporating N-doped carbon without Cu_1_ doping under electrofiltration conditions. Experiments were performed in the filtration system using a feed solution with 10 mg-N L^−1^ NaNO_3_ and 10 mM Na_2_SO_4_ at a current density of 2.5 mA cm^−2^. Error bars represent SDs from triplicate measurements. (*B*) EPR spectra of the electrofiltration system at different voltages using DMPO (50 mM) as the spin-trapping agent. −3.5 V and −1.5 V (vs. Ag/AgCl) represent the voltages with and without significant contributions from the hydrogen evolution reaction, respectively, during the NO_3_RR. Asterisks (*) indicate the characteristic peaks of H*. Mass density distributions of NO under different collision frequency distributions, estimated through molecular dynamics simulation, when the molecules (*C*_*1*_) transport to the surface and (*C_2_*) penetrate through the surface, where the surface possesses a Cu−N_4_ moiety-doped carbon (Cu−N_4_/C) structure. Low (representing flow-by mode) and high (representing flow-through mode) collision frequency distributions were determined considering collision probability mean values of 0.4 and 0.8, respectively.

Flow-through electrofiltration using the Cu_1_/NC@CNT-FEM improves the NO_3_^−^ reduction selectivity to N_2_, while enhancing the reaction kinetics compared with flow-by operation mode (Fig. 2*C*). We confirm the central role of the Cu_1_ for enabling the NO_3_RR during electrofiltration by comparing the reduction performance of the Cu_1_/NC@CNT-FEM (green shading) with a CNT-FEM incorporating N-doped carbon without Cu_1_ doping (gray shading), as shown in Fig. 3*A*. The membrane incorporating Cu_1_ shows significantly higher activity (97% NO_3_^−^ conversion) compared with the metal-free CNT-FEM (<10% NO_3_^−^ conversion).

The interactions among the in situ generated hydrogen species, the intermediate reduction species, and the catalysts might potentially affect NO_3_^−^ reduction activity and N_2_ selectivity under electrofiltration in addition to the mass transport enhancement effect. We first investigate the influence of hydrogenation mechanisms, driven by H* and/or direct electron transfer, on the rate and selectivity of electrochemical NO_3_RR. (19, 30) Understanding the hydrogenation mechanisms is necessary to gain deeper insight into how electrofiltration modifies the reactions. To explore the hydrogenation mechanisms involved in the NO_3_RR during flow-through operation, we controlled the relative contributions of various hydrogen species, including H^+^, H_2_, and H*.

Upon increasing the H^+^ concentration using a feed solution with initial pH of 3.0, the membrane shows similar NO_3_^−^ removal efficiency and N_2_ selectivity compared with the control (Fig. 3*A*), indicating a limited impact of H^+^ on NO_3_^−^ reduction during electrofiltration. In fact, the pH of the permeate is maintained at a high value of ~11.0 during the entire operation when treating the acidic feed solution (*SI Appendix*, Fig. S12). The ability of the membrane to modify the local pH can result in an even higher alkalinity in the pores, (34) suppressing the hydrogenation of NO_3_^−^ by direct electron transfer using H^+^. H_2_ generated from water electrolysis is another hydrogen source that may contribute to NO_3_^−^ reduction. The contribution of H_2_ to NO_3_^−^ reduction is investigated by applying surface flushing for removing the H_2_ bubbles from the membrane (see schematics in *SI Appendix*, Fig. S13). As a result, the NO_3_^−^ removal efficiency and N_2_ selectivity decrease significantly to 64% and 68%, respectively, demonstrating the vital contribution of H_2_ to the NO_3_RR during electrofiltration.

The effect of H* on NO_3_^−^ reduction during electrofiltration is investigated by conducting a radical quenching experiment using *tert-*butanol (*t*-BuOH) as the scavenger (Fig. 3*A*). The reduced NO_3_^−^ conversion and increased formation of NH_4_^+^ and nitrite (NO_2_^−^) in the presence of the scavenger suggest that H* is involved in NO_3_^−^ reduction and N_2_ generation. We further support this result by increasing the initial NO_3_^−^ concentration to obtain a relatively low ratio of available H* to NO_3_^−^, leading to a decline of the N_2_ selectivity (*SI Appendix*, Fig. S14). It has been suggested in the literature that mismatch in the supply and demand of H* hinders N_2_ generation during electrochemical NO_3_^−^ reduction, which may explain the observed lower N_2_ selectivity for relatively high nitrate concentrations. (41) Electron paramagnetic resonance (EPR) analysis enables direct measurement of the electrogeneration of H* in the Cu_1_/NC@CNT-FEM. The characteristic peaks for H* can be observed only when the hydrogen evolution reaction occurs (Fig. 3*B*), suggesting that the membrane does not produce H* directly through H^+^. These results demonstrate that matching the relative amount of H*, generated primarily by H_2_ dissociation, to the concentration of NO_3_^−^ is critical to controlling selective N_2_ generation for NO_3_^−^ reduction during flow-through electrofiltration. In particular, the dense, interwoven membrane structure may be able to trap the electrogenerated H_2_ by the downward water flow, promoting in situ conversion of H_2_ to H* and enabling a high density of localized H* within the membrane to facilitate NO_3_^−^ reduction to N_2_.

In addition to matching the amount of H* for NO_3_^−^ reduction to N_2_, electrofiltration might also facilitate NO_3_^−^ reduction by modifying the interaction between the intermediate reduction species and the catalysts. In the electrochemical NO_3_RR, the key adsorbed intermediate that serves as the divergent center toward N_2_ and NH_3_ generation is NO. (42, 43) It has been demonstrated that electrocatalysts with Cu−N_4_/C structure favor the formation of N−N bonds by combining one adsorbed NO molecule with one solvated NO molecule. (27) Thus, we applied molecular dynamics (MD) simulations to study the adsorption and transport processes of NO under high (represents flow-through mode) and low (represents flow-by mode) molecular collision frequencies using a substrate constructed with a Cu−N_4_/C structure to simulate the Cu_1_/NC catalyst surface.

As shown in Fig. 3*C_1_*, the closer peak position to the substrate and the stronger peak intensity under high collision frequency (*Right* side) suggest that the rate of transport of NO molecules to the catalyst surface is greater in flow-through mode compared with flow-by mode, therefore facilitating NO adsorption. The enhanced NO adsorption under high collision frequency should be favorable for N_2_ formation because of the greater density of adsorbed NO-containing intermediates during the hydrogenation process (see reaction pathway in *SI Appendix*, Fig. S15). Additionally, the greater collision frequency can also increase NO_2_^−^ transport to accelerate the formation of NO (*SI Appendix*, *Left* panel in Fig. S16). Furthermore, the MD simulations predict greater penetration of NO through the Cu_1_/NC surface in flow-through mode (representing greater adsorption to the surface) compared with flow-by mode, as indicated by the greater distance between the peak position and the substrate under high collision frequency (*Left* side in Fig. 3*C_2_*). Similarly, NO_2_^−^ penetration can also be enhanced under flow-through mode (*SI Appendix*, *Right* panel in Fig. S16). The greater collision frequencies of solvated NO and NO_2_^−^ molecules under flow-through mode could enhance the probability of adsorption to catalyst active sites, enriching the concentration of adsorbed reaction intermediates and enhancing the reaction rate and selectivity for N_2_ formation.

In sum, flow-through electrofiltration matches the relative amount of H*, produced from in situ generated H_2_, for reducing NO_3_^−^ to N_2_, and enables a greater probability of adsorption of NO as well as NO_2_^−^ to the catalyst surface in addition to mass transport enhancement. These specific mechanisms are crucial to accelerating electrochemical NO_3_^−^ reduction and enhancing the selectivity of the Cu_1_ catalyst toward N_2_ generation during electrofiltration.

## Discussion

**Fig. 4.**
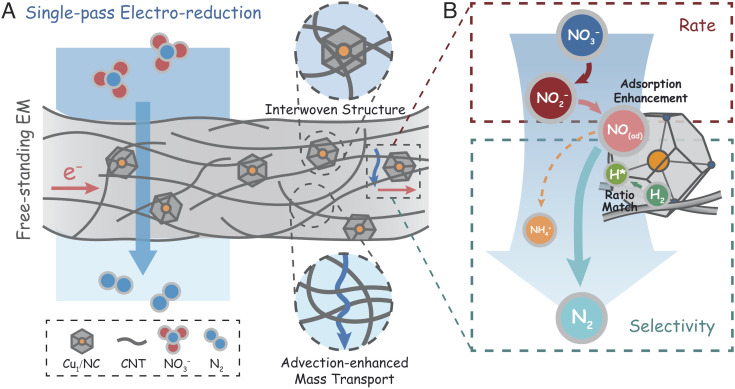
Proposed mechanisms for highly efficient and selective electrochemical nitrate reduction using the Cu_1_/NC@CNT-FEM. (*A*) Schematic describing the structural characteristics and properties of the free-standing electrified membrane (EM) during single-pass electroreduction of NO_3_^−^, including the immobilization of catalysts in the CNT interwoven structure and advection-enhanced mass transport. (*B*) Schematic illustrating the mechanism for achieving highly efficient and selective NO_3_^−^ reduction to N_2_ under flow-through electrofiltration, including enhancing the adsorption of NO and matching the supply of H* through H_2_ dissociation.

The facile triggering of harmful algal blooms by ultra-low concentrations of NO_3_^−^ motivates the development of technologies for efficient NO_3_^−^ destruction. In this study, we fabricate a free-standing carbonaceous electrified membrane (EM) via incorporating copper single-atom (Cu_1_) catalysts into a CNT interwoven framework, achieving near-complete removal of ultra-low concentration NO_3_^−^ (10 mg-N L^−1^) with high N_2_ selectivity in a single-pass electrofiltration with residence time on the order of seconds. Proposed mechanisms involved in this highly efficient and selective NO_3_^−^ reduction process using the membrane are shown in Fig. 4.

The CNT interwoven configuration can trap the Cu_1_ catalysts in the CNT framework while maintaining the structural stability and electrical conductivity of the membrane (Fig. 4*A*). This unique structure enhances NO_3_^−^ reduction activity during electrofiltration based on advection-enhanced mass transport. Advective transport of water through the EM decreases the thickness of the diffusional boundary layer to the length scale comparable to the pore radius in flow-through operation mode, (32) significantly enhancing the mass transport rate.

Fig. 4*B* shows two key steps in electrochemical NO_3_^−^ reduction: NO_2_^−^ formation is the rate-determining step (red box) and intermediate NO reduction is the selectivity-determining step (cyan box). Copper is the most commonly employed catalyst material for electrochemical NO_3_^−^ reduction to NO_2_^−^, owing to its high activity for catalyzing the first charge transfer in the rate-determining step. (30) However, the mismatch between the adsorption of NO and the availability of H* hinders the subsequent N_2_ formation in the selectivity-determining step. In addition to mass transport enhancement, the application of flow-through electrofiltration could accelerate NO_3_^−^ removal and facilitate N_2_ generation primarily through i) enhancing the adsorption probability of the NO as well as NO_2_^−^ molecules to the catalyst surface, ii) increasing the collision frequency between the adsorbed and solvated NO-containing intermediates, and iii) matching the relative amount of H* through conversion of in situ generated H_2_, where H* is the primary species driving the hydrogenation.

We demonstrate the feasibility and structural advantages of utilizing flow-through electrofiltration to achieve highly efficient and selective reduction of low-concentration NO_3_^−^ in the absence of chlorination. Platinum group metals are commonly introduced to steer the NO_3_^−^ reduction selectivity toward N_2_ by promoting the adsorption of the intermediate reduction species, such as NO and NO_2_^−^, and the generation of H*. (19, 42) Additionally, porous electrodes with new configurations, such as three-dimensional electrodes (44, 45) and open-framework electrodes, (41) have also been designed to enhance mass transport and to balance the supply and demand of H* for N_2_ generation. Flow-through electrofiltration using the EM realizes similar mechanisms without requiring precious platinum group metals, and the residence time (10 s) and energy consumption per order (*E*_EO_, 2.0 kWh m^−3^) are much lower compared with nonmembrane porous electrodes operated in flow-by systems (1 to 5 h and 7.2 to 18.5 kWh m^−3^, respectively).

We emphasize that residual concentration is a crucial index for estimating the feasibility of the NO_3_^−^ removal technologies in practical applications for preventing contamination of drinking water sources and eutrophication in aquatic environments. Harmful algal blooms can be triggered by ultra-low nitrogen concentrations, whereas weak mass transport under such concentrations hinders the efficient and near-complete removal of NO_3_^−^. The low reactant concentrations in feedwaters inevitably lower the reaction rate and favor competitive side reactions in electrochemical processes, decreasing the current efficiency. (46, 47) We note that the membrane can achieve a Faradaic efficiency of 80% with high NO_3_^−^ removal efficiency in a single-pass electrofiltration when treating water with initial NO_3_^−^ concentration at municipal wastewater levels (50 mg-N L^−1^, *SI Appendix*, Fig. S17). The treatment performance of the membrane could be potentially optimized by reducing the pore size as well as by increasing the catalyst loading. In addition, the small amount of generated NH_4_^+^ (<2 mg-N L^−1^) in the treated water can be removed in a subsequent chlorination process with a typical chlorine dosage of 6 to 15 mg-Cl_2_ L^−1^ for water disinfection. (48) However, the application of chlorination to remove NH_4_^+^ with a higher concentration can significantly increase the potential for harmful chlorinated by-product formation. (49, 50)

The application of flow-through EMs can be a viable alternative to address the challenges involved in conventional flow-by electrochemical systems, such as limited mass transport and catalyst utilization efficiencies. Low-pressure membrane filtration is one of the most widely applied technologies for water purification and wastewater treatment. (51) EMs could further extend the performance of membrane technologies beyond pure separation by introducing electroactivity as an additional function. The compact and modular construction enables EMs to be easily integrated into existing water treatment systems, such as drinking water and municipal wastewater treatment plants, or to serve as point-of-use devices for a broader range of water treatment applications (52).

In this study, we demonstrate the feasibility of using EMs to modify the pathways and kinetics of the NO_3_RR during electrofiltration. Future efforts could focus on tailoring electrocatalysts with specific functions during flow-through operation, since the catalyst designs at present are more appropriate for flow-by systems. Additionally, the CNT interwoven framework also provides a promising platform for integrating other electrocatalysts into EMs to realize additional functions during flow-through operation of electrified water treatment. Since the EMs are free-standing, they may be fabricated in various configurations such as in hollow-fiber membranes, enabling facile integration with existing water treatment systems and benefiting from existing membrane technological optimization.

## Materials and Methods

### Synthesis of Cu_1_/NC.

Typically, 0.2-g Cu(NO_3_)_2_·2.5H_2_O and 3.0-g Zn(NO_3_)_2_·6H_2_O were dissolved in 40 mL methanol, followed by the addition of 80 mL methanol containing 6.5 g 2-methylimidazole. After stirring the solution at room temperature for 12 h, the obtained Cu-doped ZIF-8 were separated by centrifugation, followed by washing with methanol three times and drying at 60 °C for 12 h. The Cu_1_/NC catalysts were prepared through pyrolysis of the Cu-doped ZIF-8 precursors at 900 °C under argon atmosphere for 3 h with a heating rate of 5 °C min^−1^. The obtained catalysts were then bleached by 1 M H_2_SO_4_ solution at 90 °C for 12 h to remove the unstable species, followed by washing with DI water until neutral and drying at 60 °C for 12 h. The content of Cu in the Cu_1_/NC was 0.81 wt%, quantified using inductively coupled plasma mass spectrometry (ICP-MS, ELAN DRC-e, PerkinElmer). Cu_NP_/NC and N-doped carbon (see SEM images in *SI Appendix*, Fig. S18) were synthesized through the same procedure but with 0.8-g Cu(NO_3_)_2_·2.5H_2_O or without Cu(NO_3_)_2_·2.5H_2_O addition.

### Fabrication of Cu_1_/NC@CNT-FEM.

Pristine CNTs were obtained by acid treatment of the as-received CNTs, as described in *SI Appendix*. Cu_1_/NC and CNTs were dispersed in a PAN solution (0.1 wt% in dimethylformamide, DMF) at 5 mg mL^−1^ with a mass ratio of 1:1, followed by sonication for 10 min using an ultrasonic probe. The as-prepared suspension was vacuum filtered onto a ceramic membrane substrate (0.45 μm, Sterlitech) with a total material loading of 100 mg. The obtained membrane was then rinsed with DI water followed by drying at 90 °C for 12 h. The Cu_1_/NC@CNT-FEM (effective area of 12.6 cm^2^) was obtained by peeling the carbonaceous layer off from the ceramic membrane substrate after drying. Cu_NP_/NC@CNT-FEM, CNT-FEM, and Cu_1_/NC-CM were fabricated through the above method but with the replacement of the Cu_1_/NC by Cu_NP_/NC with same Cu mass loading, without Cu_1_/NC addition, or without CNT addition and active layer separation.

### Catalyst and Membrane Characterization.

To characterize the Cu_1_ structure of the Cu_1_/NC catalysts, X-ray absorption spectroscopy (XAS) of the Cu *K*-edge was measured at Beamline 8-ID of the National Synchrotron Light Source II (Brookhaven National Laboratory) using a Si (111) double-crystal monochromator and a passivated implanted planar silicon fluorescence detector. XANES data were collected at room temperature with energy calibration using a copper foil. The morphology of Cu_1_ was determined using HAADF-STEM (Titan Cubed Themis G2 300, FEI). SEM (SU8230, Hitachi) coupled with EDS (XFlash 5060FQ, Bruker) was employed to investigate the surface and cross-section morphologies and elemental distribution of the Cu_1_/NC@CNT-FEM. Water contact angles were measured by the sessile drop method using a contact angle goniometer (OneAttension, Biolin Scientific). Water flux was calculated by dividing the permeate flow rate by the effective membrane area. Additional methods employed for characterizing the catalysts and the membrane are detailed in *SI Appendix*.

Electrochemical measurements were performed by an electrochemical workstation (CHI 660E, CH Instruments) in a typical three-electrode electrochemical cell containing the working electrode, a RuO_2_-IrO_2_/Ti mesh as the counter electrode, and an Ag/AgCl electrode as the reference electrode. EIS of the Cu_1_/NC@CNT-FEM, CNT-FEM, and Cu_1_/NC-CM were conducted by applying frequencies ranging from 1 to 10^6^ Hz in a 10 mM Na_2_SO_4_ solution at open circuit voltage. CV curves of the Cu_1_/NC and CNTs were collected at a scan rate of 20 mV s^−1^ in an electrolyte containing either 100 mM Na_2_SO_4_ or 100 mM Na_2_SO_4_ with 100 mg-N L^−1^ NaNO_3_ using glassy carbon as the support.

### Electrofiltration Experiments.

Electrofiltration experiments were performed using a cross-flow membrane filtration system. The electrofiltration cell consisted of a feed chamber and a permeate chamber (*SI Appendix*, Fig. S19). The Cu_1_/NC@CNT-FEM and a RuO_2_-IrO_2_/Ti mesh were placed in the cell with 1-cm spacing, serving as the cathode and anode, respectively. Unless otherwise noted, an electrolyte containing 10 mg-N L^−1^ NaNO_3_ and 10 mM Na_2_SO_4_ was used as the feed solution. The feed (500 mL) was circulated at a flow rate of 200 mL min^−1^ by a peristaltic pump and a transmembrane pressure of ~0.25 bar was applied to obtain a permeate flow rate of 1 mL min^−1^ (corresponding to residence time of 10 s) (*SI Appendix*, Fig. S20). NO_3_^−^, NO_2_^−^, and NH_4_^+^ concentrations in both the permeate and the feed were quantified after 1 h of operation. The concentrations of NO_3_^−^, NO_2_^−^, and NH_4_^+^ were determined according to the cadmium reduction method, the diazotization method, and the Nessler method, respectively, using assay kits (HI93728 for NO_3_^−^, HI93707 for NO_2_^−^, and HI93715 for NH_4_^+^, Hanna Instruments). Ion chromatography (IC, 930 Compact, Metrohm) was also employed to double-check the concentrations of the nitrogen ions.

### Investigation of the Effect of Hydrogen Species on Nitrate Reduction.

We investigated the effect of different hydrogen species including H^+^, H_2_, and H* on NO_3_^−^ reduction during electrofiltration. The effect of H^+^ was evaluated by adjusting the initial pH of the feed solution to 3.0 using 1 M H_2_SO_4_ solution. Surface flushing was applied by increasing the cross-flow rate to 1,000 mL min^−1^ and lowering the flow orientation close to the membrane surface to remove the electrogenerated H_2_ bubbles on the membrane. *t*-BuOH (200 mM) was injected into the feed for quenching H*. The generation of H* through the Cu_1_/NC@CNT-FEM was measured using EPR spectroscopy (EPR-300E, Bruker EleXsys) with DMPO (50 mM) as the spin-trapping agent.

### Molecular Simulations.

We applied first-principles MD methods to study the molecular adsorption and transport processes of NO and NO_2_^−^ under different molecular collision probabilities using the CP2K/Quickstep software package. Surfaces consisting of Cu_1_ doped in a graphite sheet with coordination environments of pyridinic and pyrrolic nitrogen atoms were constructed as the substrate. The MD algorithm combined the NVT ensemble and Nosé–Hoover thermostat, with 1-fs step size and 200,000 steps. The force and velocity were calculated using density functional theory (DFT) with the B3LYP functional, Gaussian-type basis set, and Goedecker–Teter–Hutter pseudopotentials combined with Gamma *k*-point and 400 eV cutoff energy. The dynamic process was carried out using energy minimization to balance the system and the Maxwell distribution method to provide the different collision probabilities of the molecules. A discrete solvent model was used to consider the role of water in the simulations. Low (represents flow-by mode) and high (represents flow-through mode) collision frequency distributions were determined considering collision probability mean values of 0.4 and 0.8, respectively, resulting in a 241 times difference between the two distributions (*SI Appendix*, Fig. S21).

## Supplementary Material

Appendix 01 (PDF)

## Data Availability

The data can be found in Harvard Dataverse (https://doi.org/10.7910/DVN/CWW6BO) (53). Other study data are included in the article and/or *SI Appendix*.
