# Supplementary material for: Free-standing membrane incorporating single-atom catalysts for ultrafast electroreduction of low-concentration nitrate
Source: Proc Natl Acad Sci U S A. 2023 Mar 6;120(11):e2217703120. doi: 10.1073/pnas.2217703120 (PMC10089203; doi:10.1073/pnas.2217703120)
Supplement: Supplementary file 1 — Appendix 01 (PDF) [file pnas.2217703120.sapp.pdf]

PNAS

Supporting Information for

Free-standing membrane incorporating single-atom catalysts for ultrafast electro-reduction of low-concentration nitrate

Xiaoxiong Wang,[a] Xuanhao Wu,[a,b] Wen Ma,[a,c] Xuechen Zhou,[a,d] Shuo Zhang,[a,e] Dahong Huang,[a,f] Lea R. Winter,[a,*] Jae-Hong Kim,[a] Menachem Elimelech [a,*]

[a] Department of Chemical and Environmental Engineering, Yale University, New Haven, Connecticut 06520, United States

[b] Department of Environmental Engineering, Zhejiang University, Hangzhou 310058, China

[c] Department of Chemical and Biotechnology Engineering, Université de Sherbrooke, Sherbrooke QC J1K 2R1, Canada

[d] Department of Civil and Environmental Engineering, The Pennsylvania State University, University Park, Pennsylvania 16802, United States

[e] College of Environmental Science and Engineering, Ministry of Education Key Laboratory of Pollution Processes and Environmental Criteria, Tianjin Key Laboratory of Environmental Remediation and Pollution Control, Nankai University, Tianjin 300350, China

[f] School of Environment and Civil Engineering, Dongguan University of Technology, Dongguan, Guangdong 523808, China

***Corresponding authors:** Lea R. Winter and Menachem Elimelech

Email: lea.winter@yale.edu; menachem.elimelech@yale.edu

This PDF file includes:

Supporting text
Figures S1 to S21
Tables S1 to S2

Supporting Information Text

Chemicals and Materials. Carbon nanotubes (CNT, multi-walled, OD × L: 6–13 nm × 2.5–20 μm, >98%), copper nitrate hemi(pentahydrate) ($Cu(NO_3)_2 \cdot 2.5H_2O$, ≥98%), N,N-dimethylformamide (DMF, ≥99.8%), 2-methylimidazole (≥99%), polyacrylonitrile (PAN), sodium nitrate ($NaNO_3$, ≥99.0%), sodium sulfate (Na_2SO_4, ≥99.0%), *tert*-butanol *(t*-BuOH, ≥99.0%), and zinc nitrate hexahydrate ($Zn(NO_3)_2 \cdot 6H_2O$, ≥98%) were purchased from Sigma-Aldrich (St. Louis, MO, USA). Hydrochloric acid (HCl, 36.5–38.0%), methanol (≥99.8), and sulfuric acid (H_2SO_4, 95.0–98.0%) were purchased from J.T. Baker (Phillipsburg, NJ, USA). 5,5-Dimethyl-1-pyrroline N-oxide (DMPO, >97%) was purchased from TCI Chemicals (Tokyo, Japan). Deionized (DI) water was obtained from a Milli−Q system (Millipore, Billerica, MA, USA).

Acid Treatment of CNTs. The as-received CNTs were pre-treated in a concentrated HCl solution (36.5–38.0%) at 90 °C under refluxing for 12 h, followed by washing with DI water until neutral and drying at 60 °C for 12 h before use.

Characterization of Cu_1/NC and Cu_1/NC@CNT-FEM. The morphology of the Cu_1/NC catalysts was investigated using SEM (SU8230, Hitachi) and TEM (Tecnai Osiris 200 kV, FEI). XPS was carried out using a scanning XPS microprobe (VersaProbe II, Physical Electronics) to characterize the functional groups. XRD (SmartLab, Rigaku) was conducted for crystal structure evaluation.

As for the characterization of the Cu_1/NC@CNT-FEM, membrane pore size distribution was estimated by analyzing the membrane surface SEM images using Nano Measurer software. Membrane pore volume was determined by the weight difference between a wet and dry membrane. The retention time of water within the membrane was calculated by dividing the pore volume by the permeate flow rate.

XAFS Data Fitting. Athena software was used for XAFS data processing, including conversion of raw data to μ(E) spectra, background subtraction and normalization, and Fourier transformation and plotting. Artemis software was used for analysis of EXAFS data using theoretical standards, including setting the range of the Fourier transform from *k*-space and fitting range parameters in *R*-space. Interatomic distance, *R*, is the bond length between central atoms and surrounding coordination atoms. The Debye-Waller factor, σ^2, represents the thermal and static disorder in the absorber-scattered distance.

Batch Setup. A batch system was applied to compare the electrochemical NO_3^- reduction performance between flow-by (batch) and flow-through (filtration) mode using the Cu_1/NC@CNT-FEM. The batch system consisted of a reactor with the membrane and a RuO_2-IrO_2/Ti mesh with spacing of 1 cm. To maintain consistency with the conditions of the electro-filtration experiment, batch experiments were performed for the treatment of 60-mL feed solution (10 mg-N L^{-1} $NaNO_3$ and 10 mM Na_2SO_4) for 1 h at a current density of 2.5 mA cm^{-2} and a string rate of 500 rpm.

Electrical Energy Consumption. Energy consumption per order (E_{EO}) for NO_3^- reduction during electro-filtration was evaluated using

$$E_{EO} = \frac{U_{cell} I}{Q \log\left[\frac{C_0}{C}\right]}$$

where U_{cell} and I are the applied voltage and current, respectively, Q is the permeate flow rate, and C_0 and C are the NO_3^- concentrations before and after treatment, respectively.

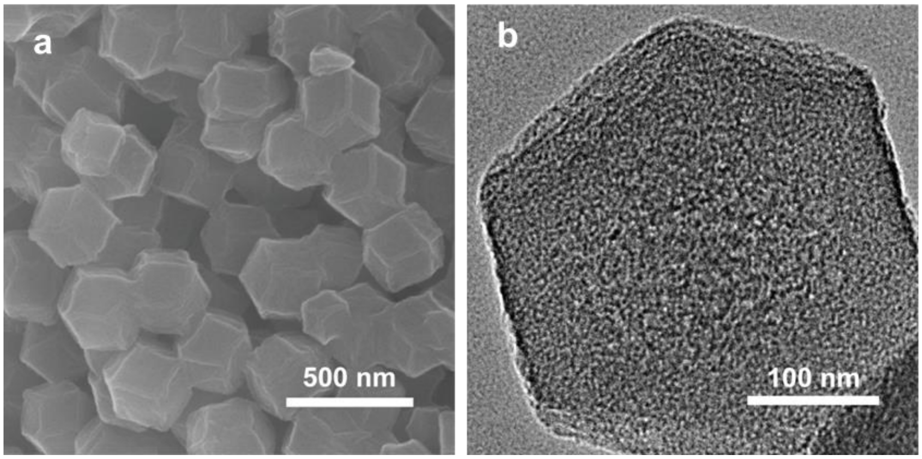

Fig. S1. (a) SEM and (b) TEM images of the Cu_1/NC.

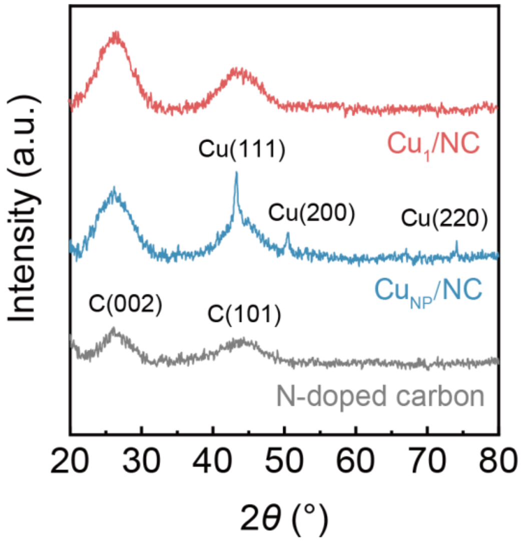

Fig. S2. XRD spectra of Cu_1/NC, Cu nanoparticles anchored on N-doped carbon (Cu_{NP}/NC), and N-doped carbon.

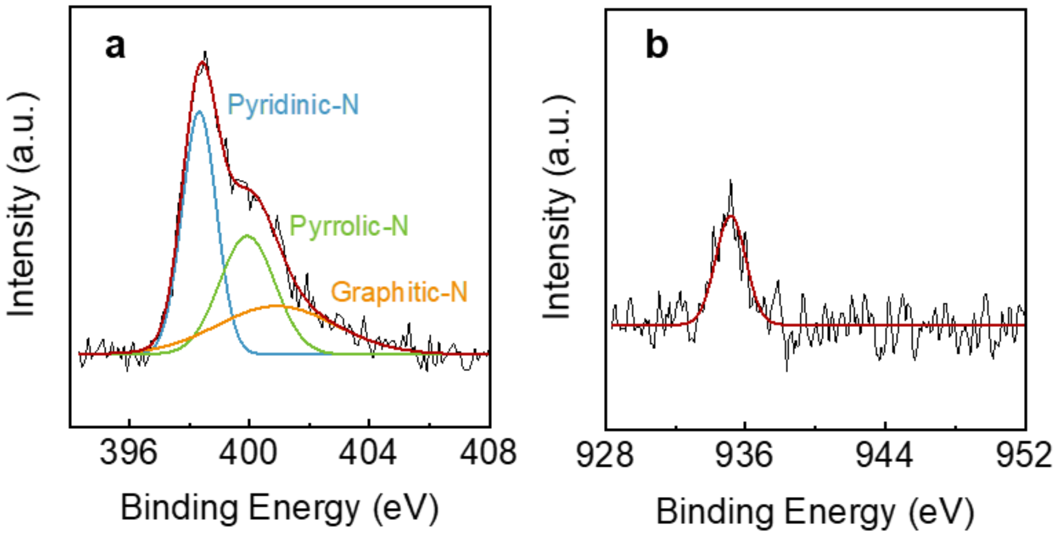

Fig. S3. XPS spectra of (a) nitrogen (N 1s) and (b) copper (Cu $2p_{3/2}$) of the Cu_1/NC.

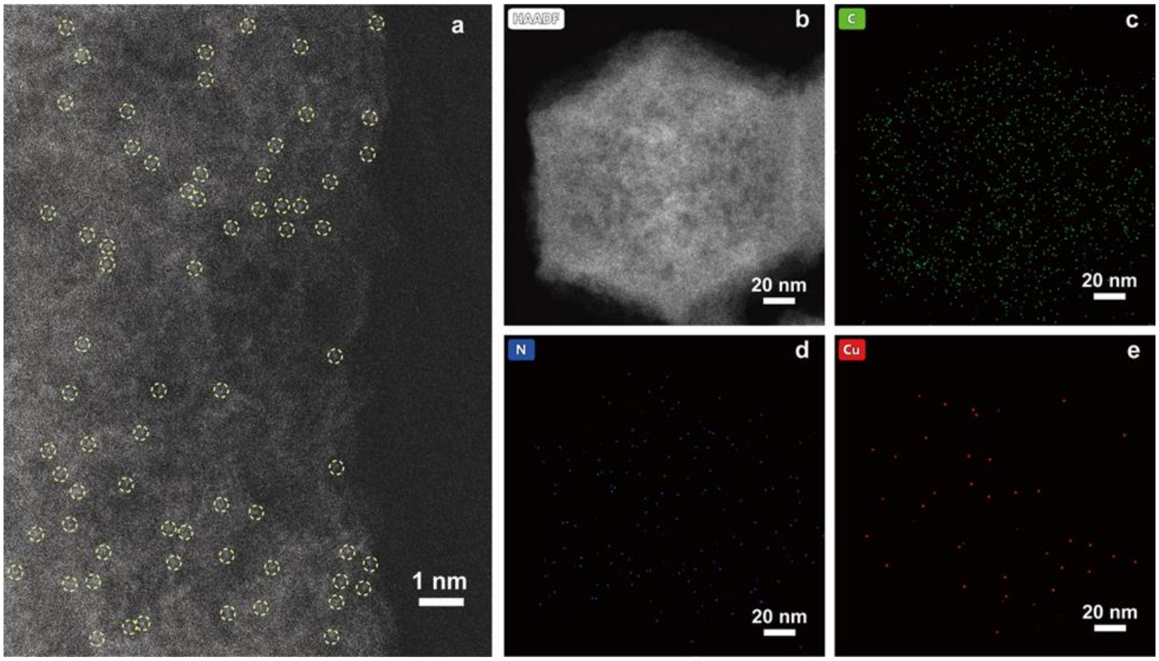

Fig. S4. HAADF-STEM images of (a) Cu_1 on N-doped carbon and (b) the Cu_1/NC catalyst and its corresponding EDS mapping of (c) C (green), (d) N (blue), and (e) Cu (red) elements. Circled bright spots indicate the Cu_1.

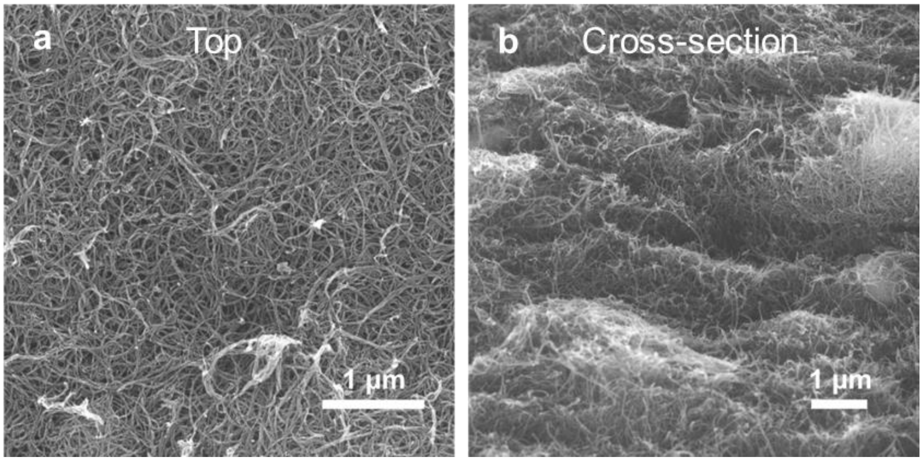

Fig. S5. SEM images of (a) top and (b) cross-section views of the CNT-FEM.

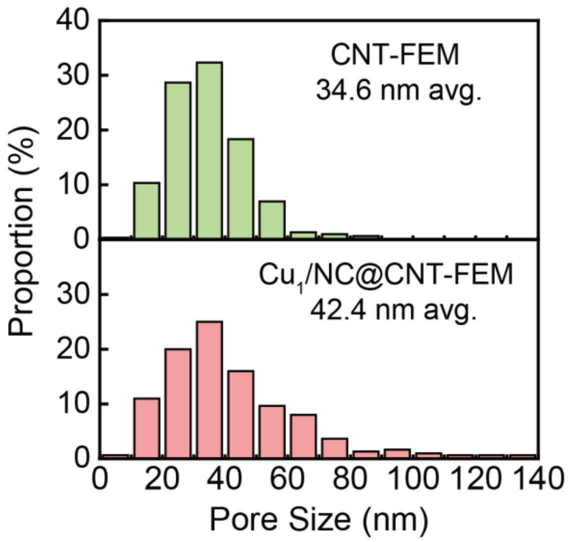

Fig. S6. Pore size distributions of CNT-FEM (top) and Cu_1/NC@CNT-FEM (bottom). Average pore sizes are indicated in the figures.

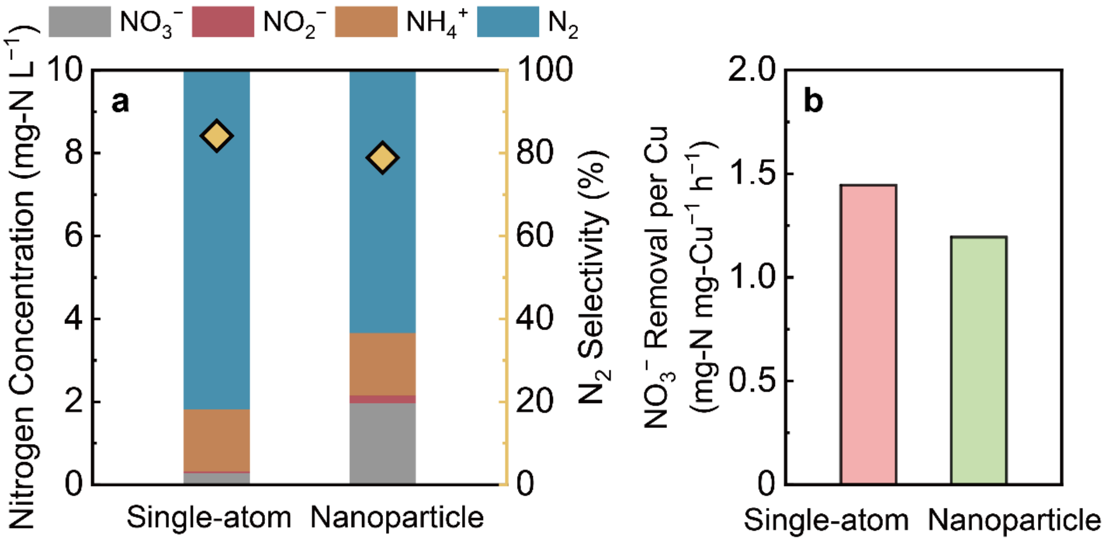

Fig. S7. Effect of applying Cu single-atom or nanoparticle catalysts on (a) NO_3^- reduction performance and (b) NO_3^- removal per mass Cu.

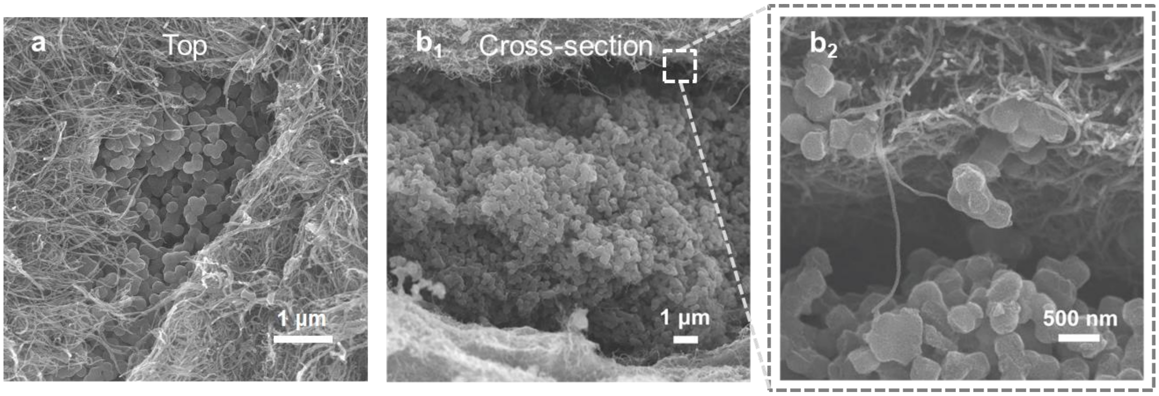

Fig. S8. SEM images of (a) top and (b) cross-section views of a Cu_1/NC incorporated CNT-FEM prepared using bath sonication for the suspension pretreatment.

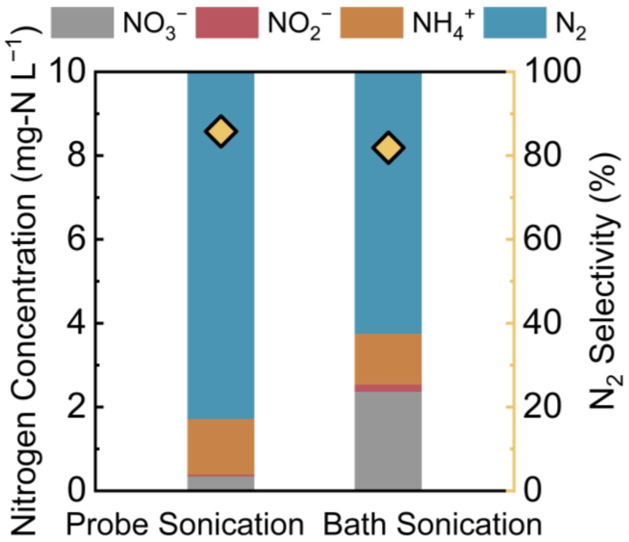

Fig. S9. Effect of sonication pretreatment method on the performance of the prepared membrane for NO_3^- reduction. The Cu_1/NC and CNT suspensions were sonicated through ultrasonic probe or bath for 10 min before membrane fabrication.

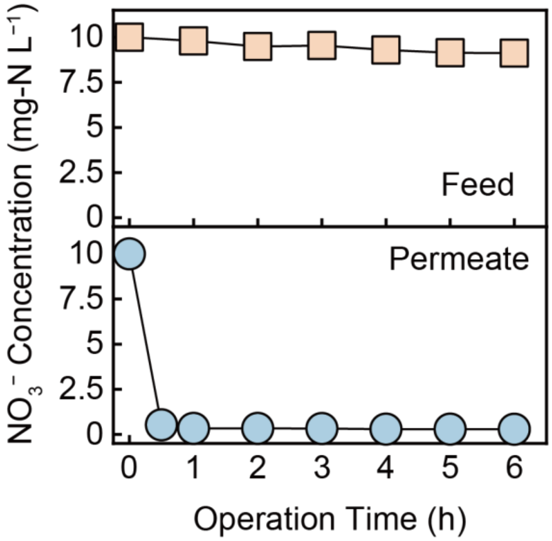

Fig. S10. Changes of NO_3^- concentration in the feed (top) and permeate (bottom) as a function of operation time. The experiment was run until the feed solution was nearly completely consumed, i.e., 6 h.

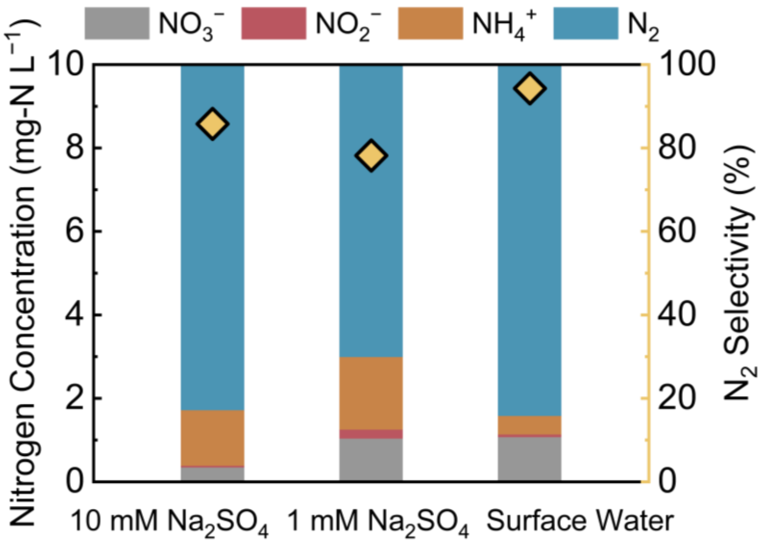

Fig. S11. NO_3^- reduction performance of the Cu_1/NC@CNT-FEM when treating different feed solutions containing 10 mg-N L^{-1} $NaNO_3$ in 10 mM Na_2SO_4, 1 mM Na_2SO_4, or simulated surface water.

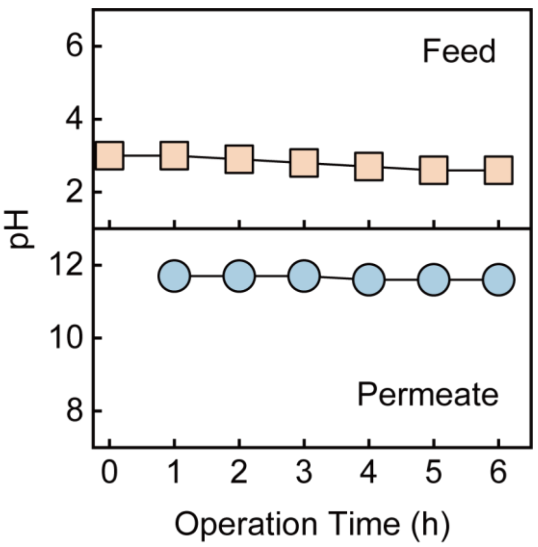

Fig. S12. Changes of pH in the feed (top) and permeate (bottom) as a function of operation time. The initial pH of the feed solution is 3.0.

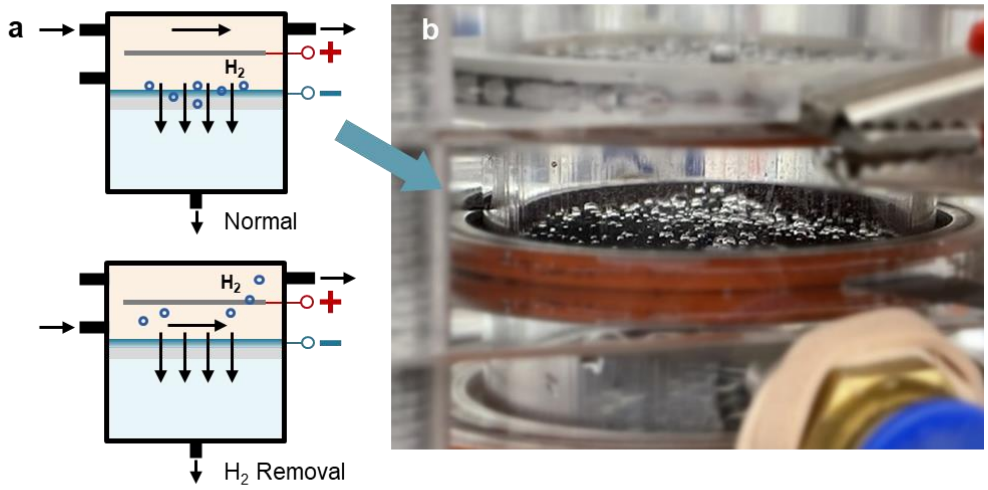

Fig. S13. (a) Schematic illustrating the surface flushing method for removing H_2 bubbles from the membrane surface. Arrows represent the direction of the water flow. Inlet flow between the anode and the membrane (bottom image, “H_2 Removal”) removes more in situ formed H_2 bubbles compared to inlet flow upstream of the anode (top image, “Normal”). (b) Photograph showing H_2 bubbles stuck on the membrane when surface flushing is not applied.

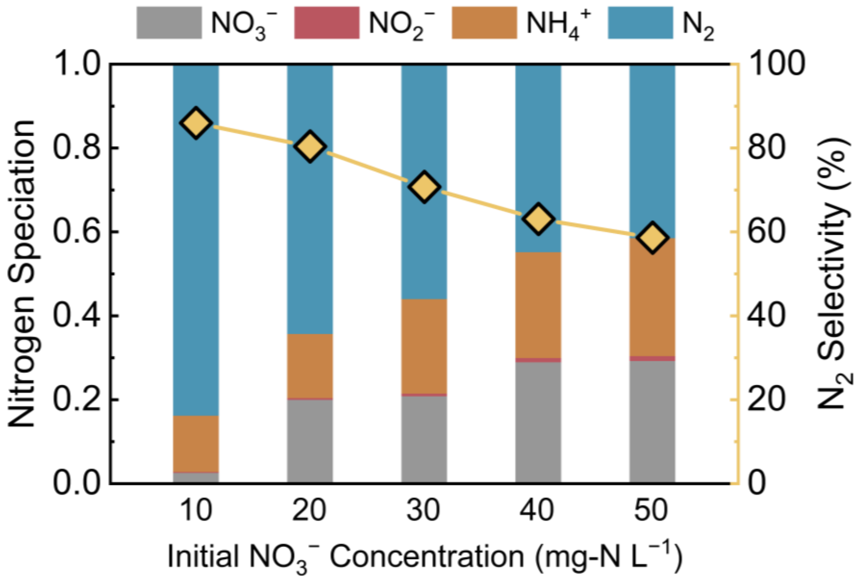

Fig. S14. Effect of initial NO_3^- concentration (10–50 mg-N L^{-1}) on NO_3^- reduction performance of the Cu_1/NC@CNT-FEM.

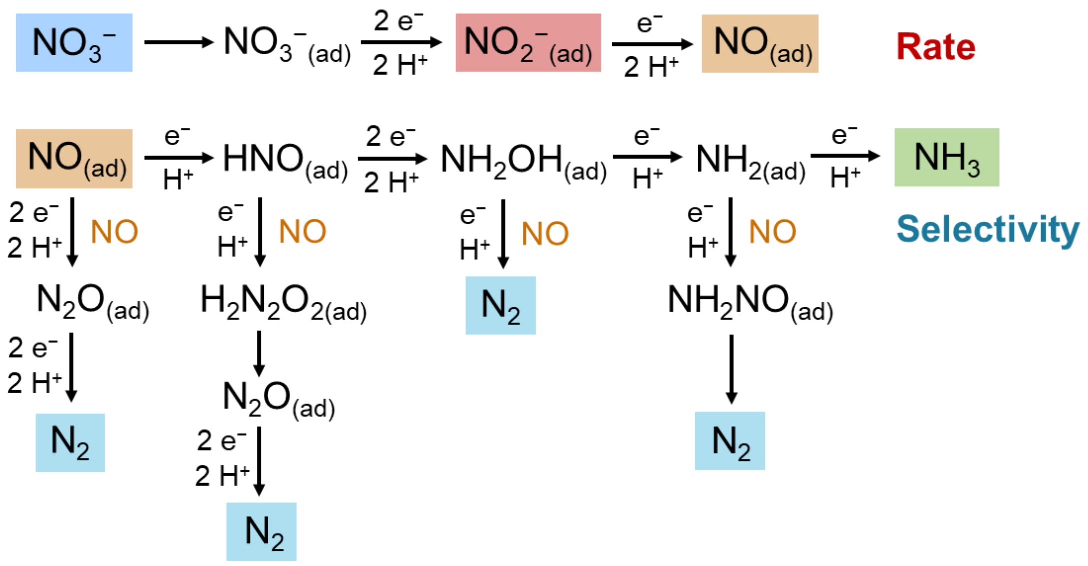

Fig. S15. Main reaction pathways involved in electrochemical NO_3^- reduction, including rate- and selectivity-determining steps. The reaction pathways are summarized according to the following references.(1, 2)

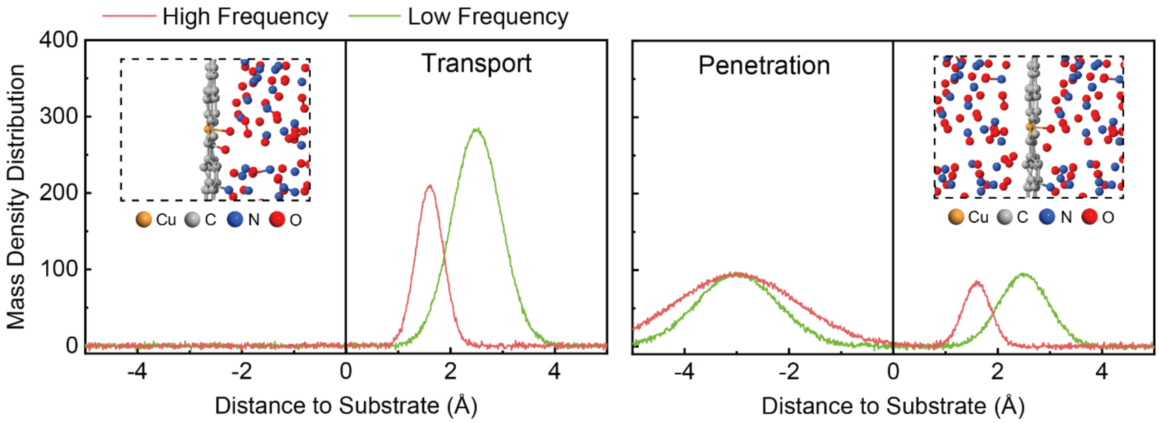

Fig. S16. Mass density distributions of NO_2^- when the molecules transport to (left) and adsorb on the surface (simulated as penetrating through the surface, right) of the Cu_1/NC substrate under different collision frequency distributions.

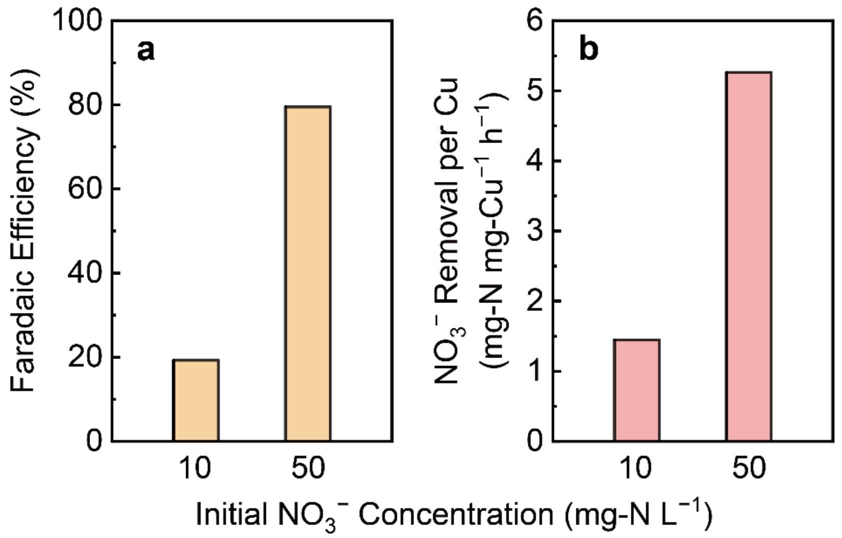

Fig. S17. (a) Faradaic efficiency and (b) NO_3^- removal per mass Cu for the Cu_1/NC@CNT-FEM using feed solutions with different initial NO_3^- concentrations of 10 and 50 mg-N L^{-1}.

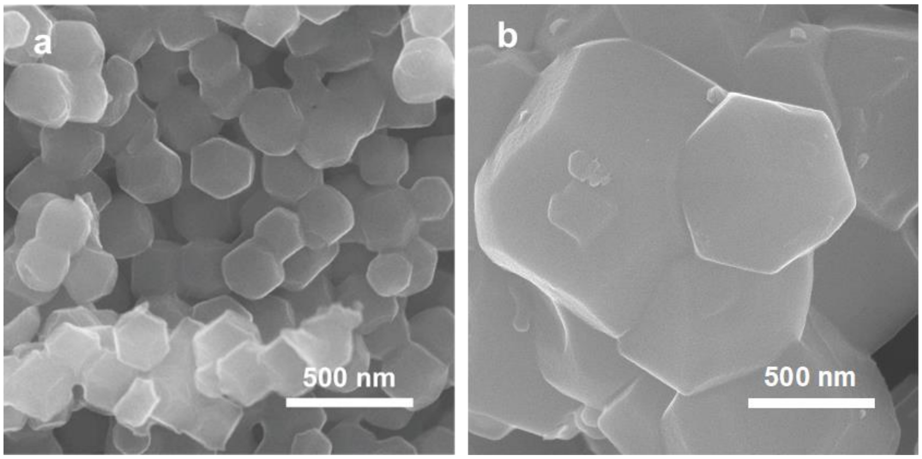

Fig. S18. SEM images of (a) N-doped carbon and (b) Cu_{NP}/NC.

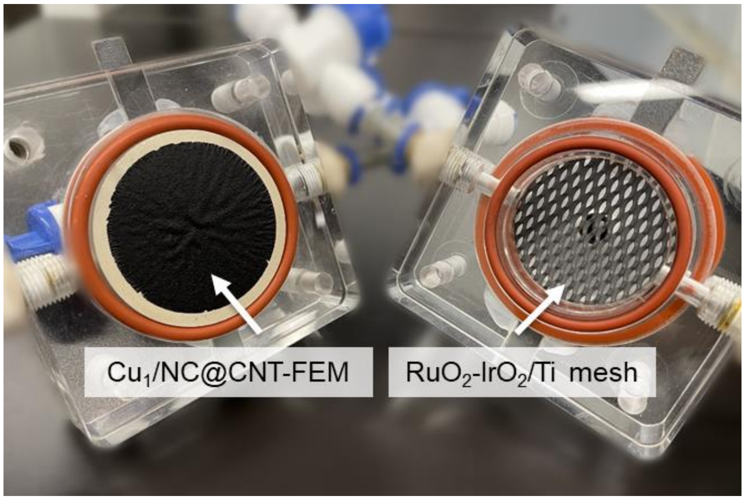

Fig. S19. Photograph of the electrified membrane filtration cell used in this study.

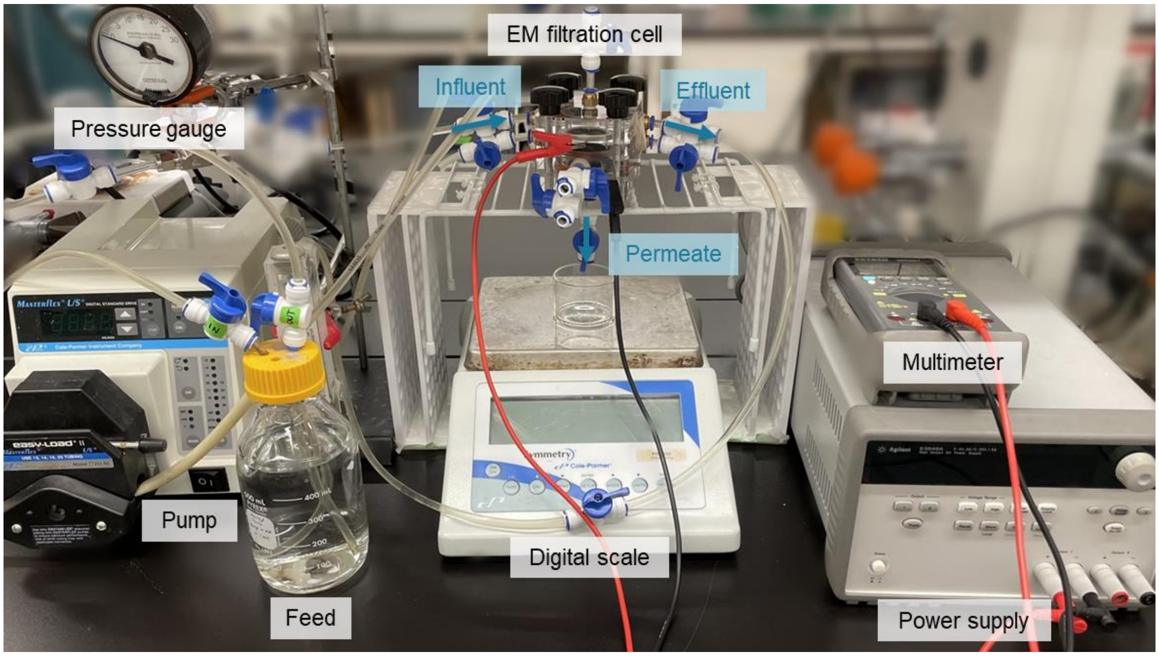

Fig. S20. Photograph of the cross-flow electro-filtration system.

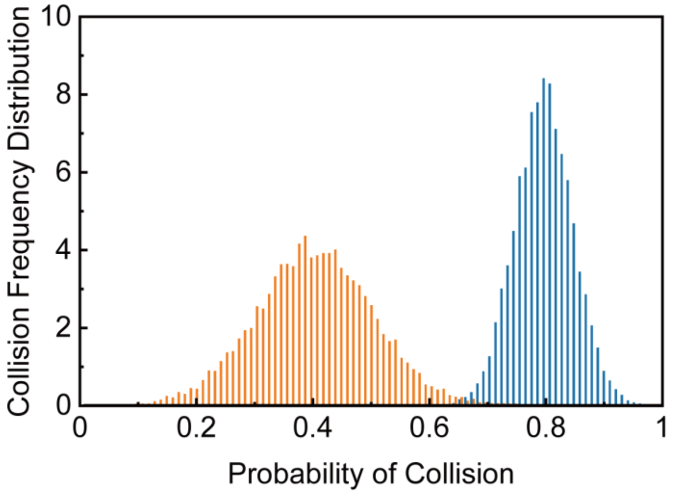

Fig. S21. Molecular collision frequency distribution under low (represents flow-by mode) and high (represents flow-through mode) collision probabilities with mean values of 0.4 and 0.8, respectively.

Table S1. Best-fit parameters extracted from the Cu *K*-edge FT EXAFS spectra.

Sample	Shell	*CN*	*R* (Å)	σ^2 (Å^2)
Cu_1/NC	Cu-N	4.4 ± 0.4	1.93	0.006
CuPc	Cu-N	4	1.96	0.009
Cu foil	Cu-Cu	12	2.54	0.009

CN: coordination number, *R*: interatomic distance, σ^2: Debye-Waller factor.

Table S2. Constituents of simulated surface water (Nanotechnology-Enabled Water Treatment Engineering Research Center standard).

Constituents	Concentration (mg L^{-1})	Concentration (mM)
Bicarbonate (HCO_3^-)	183	3.0
Calcium (Ca^{2+})	40	1.0
Chloride (Cl^-)	71	2.0
Fluoride (F^-)	1.0	0.053
Magnesium (Mg^{2+})	12	0.50
Nitrate (NO_3^-)*	44.3 (10.0 as N)	0.71
Phosphate (PO_4^{3-})	0.12	0.0013
Silica (SiO_2)	20 as SiO_2	0.33
Sodium (Na^+)	102	4.44
Sulfate (SO_4^{2-})	48	0.50
Ionic strength		9.1
pH	7.5 ± 0.25	

*Nitrate concentration is modified to be consistent in this study.

SI References

1. S. Garcia-Segura, M. Lanzarini-Lopes, K. Hristovski, P. Westerhoff, Electrocatalytic reduction of nitrate: Fundamentals to full-scale water treatment applications. *Applied Catalysis B: Environmental* **236**, 546-568 (2018).
2. Y. Wang, C. Wang, M. Li, Y. Yu, B. Zhang, Nitrate electroreduction: mechanism insight, in situ characterization, performance evaluation, and challenges. *Chemical Society Reviews* **50**, 6720-6733 (2021).
